# Comparison of the selected parameters of the anterior segment of the eye between femtosecond laser-assisted cataract surgery, microincision cataract surgery, and conventional phacoemulsification: A case-control study

**DOI:** 10.1097/MD.0000000000018340

**Published:** 2019-12-27

**Authors:** Edyta Chlasta-Twardzik, Anna Nowińska, Edward Wylęgała

**Affiliations:** Chair and Clinical Department of Ophthalmology, School of Medicine with Division of Dentistry in Zabrze Medical, University of Silesia Katowice, Poland.

**Keywords:** clinical outcomes, conventional phacoemulsification surgery, femtosecond laser-assisted cataract surgery, intraoperative parameters, manual phacoemulsification, microincision cataract surgery

## Abstract

The purpose of our study was to compare the selected parameters of the anterior segment of the eye in patients after femtosecond laser-assisted cataract surgery (FLACS) with the results of microincision cataract surgery (MICS) and conventional phacoemulsification surgery (CPS). This single-center prospective randomized comparative observational study included 87 patients. Patients were randomly selected into group A (FLACS), group B (MICS) and group C (control group). All the surgeries were performed by the same experienced surgeon. Preoperative and postoperative parameters were evaluated: best-corrected visual acuity (BCVA), endothelial cell density (ECD), endothelial cell loss percentage (ECL%), central corneal thickness (CCT), central anterior and posterior corneal astigmatism induction, posterior corneal elevation map were measured. Intraoperative parameters: effective phacoemulsification time (EPT), balanced salt solution use (BSS use), total surgical time and suction time were analyzed. Examination was performed preoperatively and on the first, seventh day, one and six months postoperatively. The follow up period was 6 months. There was no statistically significant difference in BCVA, central anterior and posterior astigmatism induction between studied groups. The ECL% was statistically significant lower in the group A on the 7th day, 1 month and 6-months postoperatively (*P* < .05). The CCT was statistically significant lower in the group A and in the group B than in the group C on the 7th postoperative day (*P* = .002). However, in the 6 months follow-up there was no statistically significant difference in the CCT between studied groups (*P* = .133). We observed statistically significant difference in change of the posterior corneal elevation map at the periphery assessed within the 90° to 120°meridian range between group A, group B and group C at every timepoint postoperatively (*P* < .05). The EPT and BSS use were statistically significant lower whilst total surgery time was statistically significant higher in the FLACS group (*P* < .05). To conclude in the 6 months follow-up there was statistically significant difference found between eyes undergoing FLACS, MICS and CPS with respect to the posterior corneal elevation map assessed within the studied range, ECL%, EPT, BSS use and total surgery time. Postoperative BCVA, central anterior and posterior astigmatism induction, CCT were comparable between studied groups.

## 1 Introduction

Cataract, along with uncorrected refractive errors, remain the leading cause of reversible blindness in the world.^[1]^ Cataract surgery is the most frequently performed ophthalmic surgery and simultaneously the most frequently performed surgical procedure in the world. Cataract surgery is considered one of the most effective and safest medical procedures in the world, as confirmed in clinical trials. The incidence of cataract increases with age. Due to the increase in life expectancy and the aging of the population, an increase in the need for cataract surgery should be expected. Phacoemulsification as introduced by Charles Kelman in the 1970s remains the standard and preferred surgical technique used in the developed world.^[2]^ Although conventional phacoemulsification (CPS) provides good visual acuity and rarely causes complications, nowadays patients expect to achieve more rapid visual rehabilitation and experience fewer traumas.

Femtosecond laser technology was initially used in corneal refractive surgery for performing LASIK flaps (Laser In-Situ Keratomileusis).^[3]^ The first human cataract surgery with the use of the femtosecond laser was performed in Europe in 2008.[^4^,^5^] The femtosecond laser (FL) has long been used in ophthalmic surgery, its use in cataract surgery is relatively new technology and still become more popular.^[6]^ Femtosecond laser has been FDA approved for the following three steps in cataract surgery: lens fragmentation using different patterns, anterior capsulotomy, self-sealing, clear corneal incisions, and arcuate incisions as well.^[7]^ Since the introduction of the femtosecond laser for cataract surgery, many research investigations have been carried out and a significant amount of publications have been published regarding its advantages and disadvantages compared to CPS. It is well-known that the time and energy of phacoemulsification directly cause the loss of endothelial cells.[^8^,^9^,^10^] Femtosecond laser pretreatment results in a significantly lower effective phacoemulsification time (EPT) compared to the standard cataract surgery thus contributing to a smaller endothelial cell loss.[^11^,^12^,^13^,^14^,^15^,^16^] Injury reduction of corneal endothelial cells contributes to shorten the recovery period and improve visual outcomes.^[17]^ Numerous studies have shown that use of FLACS leads to more accurate reproducible capsulotomy geometry compared to manual capsulorhexis.[^18^,^19^,^20^] Using the laser in cataract surgery we get a capsulotomy with an ideal shape that affects intraocular (IOL) lens position in result reduce the probability of IOL decentration and tilt.[^21^,^22^] This is important for calculating the power of implanted intraocular lens, and thus for the final refractive outcomes and visual acuity.[^17^,^23^] The many advances in cataract surgery technology have reduced the incision size to 2.0 mm or smaller. The cataract surgery in which the cut size is below 2.0 mm is called MICS. The introduction of a microincision results in better control of induced post-operative astigmatism and minimizes peri-operative corneal damage, which increases the safety of the procedure and accelerates convalescence.^[24]^

The purpose of this study was to evaluate the safety, efficacy as well as to compare the correlation between selected parameters of the anterior segment of the eye in 3 studied groups: in the group A (FLACS group), in the group B (MICS group) and in the group C (control group). Preoperative, intraoperative and postoperative data were analyzed in 6-month follow-up. Preoperative and postoperative outcomes included: best-corrected visual acuity (BCVA), endothelial cell density (ECD), endothelial cell loss percentage (ECL%), central corneal thickness (CCT), central anterior and posterior astigmatism induction and changes in posterior corneal elevation map were measured. Intraoperative outcomes included: total surgical time, effective phacoemulsification time (EPT), BSS usage and suction time.

## 2 Material and methods

This prospective randomized comparative observational study was performed in a single center at the Chair and Clinical Department of Ophthalmology of Medical University of Silesia, District Railway Hospital in Katowice, Poland. The study groups consisted of 87 eyes from 87 patients qualified for cataract surgery between September 1, 2017, and December 31, 2018. This study conformed to the tenets of the *Declaration of Helsinki* and study protocol was approved by the local Bioethical Commission at the Silesian Medical University in Katowice, Poland (Resolution No. KNW / 0022 / KB1 / 47/17 of 30/05/2017 and Resolution No. KNW / 0022 / KB1 / 47 / II / 17/18 of 25/09/2018). In accordance with the requirements of the *Helsinki Declaration*, the participants of the study were informed about the purpose, nature and method of research being carried out. Then, after expressing their written, informed consent, they were qualified for the research project. Inclusion criteria consisted of signing informed consent for participation in the study, patients with diagnosed cataracts qualified for surgical treatment an age of 50 years of older, transparent cornea, mydriasis > 5 mm, cataract grade from 2–4 (nuclear opacity 2–4 (NO2–NO4) and nuclear color 2–4 (NC2–NC4)) according to the Lens Opacities Classification System III (LOCS III), no current infections.^[25]^ Exclusion criteria consisted of no signature or withdrawal of the patient's consent to participate in the study, the age under 50 years, previous surgical interventions within the eyes, poorly dilating pupils of less than 5 mm that contraindicate femtosecond laser–assisted cataract surgery or any other defect of the pupil (e.g., iris adhesions), corneal diseases, dystrophies or other pathology (e.g., pterygium, corneal opacities or scaring, significant superficial punctuate keratitis) that precludes the transmission of laser wavelength or that distorts laser light, complicated cataracts (e.g., lens subluxation, hard cataracts, traumatic cataract, pseudoexfoliation syndrome), concomitant eye pathologies (e.g., uveitis, manifest glaucoma, strabismus, macular degeneration, diabetic retinopathy), monocular patients. Criteria for inclusion in the study and exclusion from the study in selected groups of patients are identical. Three groups were formed, based on the technique of cataract surgery. Patients were randomly selected into 3 groups: group A (FLACS group), group B (MICS group) and group C (control group-SICS). Surgical technique (FLACS, MICS or CP) was randomized using an online random number generator (https://www.randomizer.org/) using computer generated random number tables (Excel software, Microsoft Corp.). Group A included 26 eyes from 26 patients who underwent femtosecond laser-assisted cataract surgery by using the low-energy femtosecond laser Zimmer LDV Z8. Group B included 31 eyes from 31 patients who underwent microincision cataract surgery. The term “micro incision” refers to the use of a sutureless, 1.8 mm clear-cornea main incision in the superior, temporal quadrant. Group C included 30 eyes from 30 patients who underwent conventional phacoemulsification which was a control group. The term “small incision” refers to the use of a sutureless, 2.4 mm clear-cornea main incision in the superior, temporal quadrant. All the surgical procedures were performed by the same experienced surgeon. After signature of the informed consent a detailed clinical history was collected for all patients and a complete ophthalmological examination including anterior segment biomicroscopy and fundus examinations, best corrected visual acuity (BCVA), central corneal thickness (CCT), endothelial cell density (ECD), anterior and posterior corneal astigmatism and posterior corneal elevation map were carried out. Cataract density was graded clinically in the dilated eye according to the Lens Opacities Classification System III (LOCS III) ^[25]^ using slit lamp (SL 990 Digital Version; CSO; Firenze, Italy) at maximum illumination without light filtering. Nuclear opacity (NO2-NO4) and nuclear color (NC2-NC4) were used as the parameter to determine cataract density. Cataract density was graded objectively using Scheimpflug imaging (The Pentacam HR, Type 70900; Oculus Germany). BCVA was measured using Snellen visual acuity charts (with the visual acuity logMAR scale). CCT, anterior and posterior corneal astigmatism were obtained by automatic measurement with an Anterior Segment Optical Coherence Tomography (Tomey, CASIA 2, AS-OCT). Scans were taken in the automatic mode. Posterior elevation map was obtained with an Anterior Segment Optical Coherence Tomography (Tomey, CASIA 2, AS-OCT). Scans were taken in the automatic mode. Three consecutive scans of the patient's eye were acquired for each control examinations. Changes of the elevation map were assessed in 8 points within 3 circle regions automatically designated by the AC-OCT. After analyzing all postoperative scans, the place and the range of the maximum posterior elevation map changes were determined. The maximum elevation changes of the posterior corneal surface occurred in the 90 to 120-meridian range for each eye examined after cataract surgery. Then, in this area the maximum elevation value was manually assessed, which was located in the peripheral part of the posterior elevation map (within the 3rd circle). ECD was measured at the corneal center using noncontact specular microscopy (NIDEK Model CEM-530). Scans were taken in the automatic mode. All of the above-mentioned examinations performed 1 day before the surgery and follow-up examinations on the first and seventh day after the surgery and in the first month and six months after the surgery. The follow-up period in each studied group was 6 months. Intraoperative parameters effective phacoemulsification time (EPT), total surgery time, balanced salt solution use (BSS use) and suction time were collected. All surgeries in both groups as well in the control group were performed using the same phaco machine (Stellaris system, Bausch & Lomb, NY).

### 2.1 Surgical technique

All surgical procedures were performed by the same experienced surgeon using topical anesthesia drops (Alcaine, proxymetacaine 5 mg/mL). Preoperatively the pupil dilation was achieved by application of 10% phenylephrine and 0.5% tropicamide. All the patients in the group A had femtosecond laser capsulotomy and lens fragmentation in the same setting, followed by traditional phacoemulsification (Stellaris system, Bausch & Lomb, Inc.) and insertion of an IOL into the capsular bag. All patients in the group B and in the group C underwent conventional phacoemulsification surgery (Stellaris system, Bausch & Lomb, NY) and insertion of an IOL into the capsular bag. All phacoemulsification parameters were kept consistent between studied groups. The standard preoperative, intraoperative and postoperative regimens were identical in the studied groups. In this study, all FLACS and CPS were performed under the surgeon's microscope in the same operating room during the procedure.

### 2.2 Femtosecond laser-assisted technique

**Figure 1:**
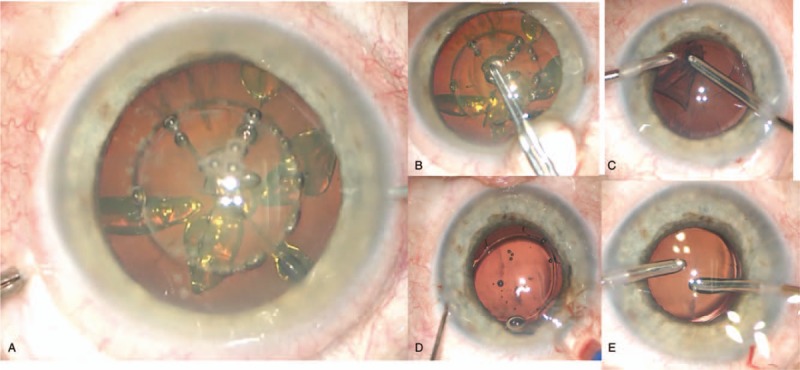
Coaxial femtosecond laser-assisted cataract surgery with the LDV Z8 system–surgical technique. A: Post-femtosecond laser treatment showing anterior capsulotomy, lens fragmentation pattern (6 segments); B: Appearance of the lens after FLACS and anterior capsulotomy removal with micro-forceps; C: Coaxial irrigation/aspiration of the residual cortex and posterior capsule; D: Placement of the intraocular lens in the capsular bag; E: removal of OVD and polishing posterior capsular bag.

FLACS was performed using Femtosecond laser ZIEMER FEMTO LDV Z8 (Fig. 1). The suction ring of a disposable liquid–patient interface was precisely docked onto the patient's eye and centered over the limbus. When the suction vacuum reached 400 mbar, the suction ring was filled with a balanced salt solution (BSS). Then the handpiece, which is attached to an articulating arm of the laser system, was docked over the corneal apex. The anterior segment structures of the eye were shown by the integrated optical coherence tomography (OCT) system which is located in the handpiece. Treatment parameters were customized individually to each patient by using the laser platform settings wizard. The energy and frequency laser pulse settings were 900mW 1 MHz for capsulotomy; 950 to 1000 mW, 2 MHz for phacofragmentation. Laser treatment started with lens fragmentation with a 6-piece pie-cut pattern, followed by an anterior capsulotomy of 5.0 mm diameter (Fig. 1A). After those procedures, the suction ring was removed from the eye surface to proceed with the phaco procedure. The femtolaser LDV Z8 is unique because unlike other FLACS systems, lens fragmentation before anterior capsulotomy is possible as the low energy results in minimal gas production, which significantly reduces the risk of intra-operative complications. The main port was positioned at 120° for the right eye and for the left eye. The main clear corneal incision was made with a 2.4 mm keratome blade by hand. Two side-ports for bimanual aspiration and irrigation tips in 1.1 mm in diameter were performed at 9 o’clock and 3 o’clock in the clear cornea for the right and left eye. The anterior chamber was filled with ocular viscoelastic device (OVD) and the anterior capsule was removed (Fig. 1B). Hydrodissection and hydrodelineation were performed. Phacoemulsification was performed using a divide and conquer technique by using Barret nuclear rotator. Residual cortex was removed using bimanual irrigation/ aspiration (Fig. 1C). Finally, a foldable intraocular lens was injected into the capsular bag (Fig. 1D). At the end of the surgery viscosurgical device (OVD) was removed, the posterior capsular bag was polished (Fig. 1E) and prophylactic intracameral cefuroxime (0,1 mL of 10 mg/mL solution) was injected. Wounds were secured by stromal hydration. The suction time, total surgical time, the effective phacoemulsification time (EPT) and balanced salt solution volume were recorded in all cases.

### 2.3 Conventional cataract surgery technique

CPS (MICS & SICS) was performed using phaco machine (Stellaris system, Bausch & Lomb, NY). In the MICS group and in the control group the corneal main incision and 2 side-ports was made in the same area as in the FLACS group. In the MICS group the main clear corneal incision was made with a 1.8 mm keratome blade by hand. In the control group, the main clear corneal incision was made with a 2.4 mm keratome blade by hand. A manual 5 mm diameter continuous curvilinear capsulorhexis with the use of cystotome was attempted under protection of an OVD. The remainder of the surgical steps required to complete the operation were identical between 3 groups. The total surgical time, the effective phacoemulsification time (EPT) and balanced salt solution volume were recorded in all cases.

### 2.4 Follow-up

Postoperative therapy for all patients in 3 groups was the same and consisted in levofloxacin drops 5 times a day for 7 days and dexamethasone eye drops 5 times a day for 7 days, followed 4 times a day for 7 days, followed 3 times a day for 7 days. All patients also received nepafenac 0.1% (Nevanac 1 mg/ml) 1 drop 3 times a day for 3 weeks. Postoperative results were evaluated at 1 day, 7 days, 1 months, and 6 months after surgery and included best corrected visual acuity (BCVA), central corneal thickness (CCT), endothelial cell density (ECD), anterior and posterior corneal astigmatism, and posterior corneal elevation map using the instruments described previously.

### 2.5 Statistical analysis

Statistical analysis of the data obtained was carried out using the R program, version 3.6.0. A *P* value < .05 was considered statistically significant. The analysis of quantitative variables was performed by calculating the mean, standard deviation, median, quartiles, minimum and maximum. The analysis of the qualitative variables was done by calculating the number and percentage the occurrence of each of the value. Comparison of the values of qualitative variables in groups was made using the Chi-square test (with Yates correction for 2×2 tables) or exact Fisher test where low expected cardinality appeared in the tables. Comparison of the values of quantitative variables in 3 groups was performed by analysis of variance ANOVA (if the variable had a normal distribution in these groups) or the Kruskal–Wallis test (otherwise). After the detection of statistically significant differences, post-hoc analysis was performed by Fisher LSD test (in the case of normal distribution) or Dunn test (in the absence of normal), in order to identify statistically significant differences between groups. The normality of the variable distribution was examined using the Shapiro–Wilk test.

## 3 Results

A total of 87 patients (24 males and 63 females) were enrolled in the study. All patients in 3 studied groups underwent a successful operation. The mean age of patients was 75.93 ± 7.65 years (range 44–91 years). Over half (72.41%) of the study population were females. The characteristics of the studied groups are reported in Table 1 including patient age, gender, and LOCS value.

**Table 1:**
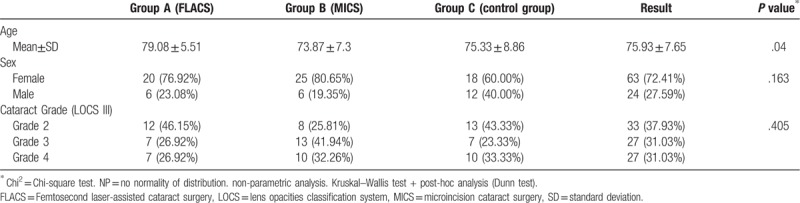
Comparison of demographic data at baseline for studied groups.

### 3.1 Intraoperative parameters

Total surgery time was significantly longer for group A compared to the group B and to the group C (14.69 ± 1.59 vs 10.12 ± 0.97 vs 10.29 ± 0.89 min; *P* < .001). The mean EPT in the group A was significantly shorter (3.91 ± 0.5 vs 4.67 ± 0.95 vs 4.69 ± 0.78 s; *P* < .001) and a reduced mean BSS usage was required (118.92 ± 20.63 vs 141.13 ± 14.93 vs 138.57 ± 15.87 mL; *P* < .001). All the results are outlined in Table 2.

**Table 2:**
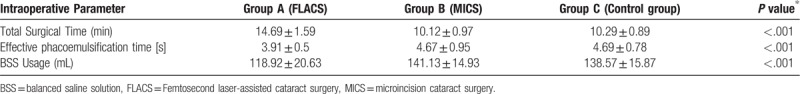
Mean intraoperative parameters in the study groups.

### 3.2 Postoperative results

Comparing the surgical techniques presented in the study a mean BCVA improvement was observed. However, the difference in the improvement of BCVA between studied groups did not reach statistical significance in 6 months follow-up (*P* > .05) (Table 3). No statistically significant differences were observed between the studied groups concerning anterior and posterior astigmatism between the baseline and postoperative values in 6 months follow-up (*P* > .05) (Table 4 and Table 5). There was a statistically significant difference between compared surgical techniques in terms of the CCT at the time point 7 days postoperatively (*P* = .002). The mean CCT in the group C (594.6 ± 28.07) was higher than in the group A (561.96 ± 35.24) and in the group B (554.26 ± 108.22) on the seventh postoperative day; however, in the 6-months follow-up not statistically significant difference was observed between 3 compared groups (*P* = .133) (Table 6). In our study, the mean ECL % of 9.32 ± 2.5 in the group A, 13.47 ± 2.13 in the group B and 13.41 ± 4.15 in the group C was observed 6 months following surgery. The ECL % was statistically significant lower in the group A compared to the group B and to the group C on the seventh postoperative day (*P* = .005), 1 month after surgery (*P* = .002) and 6 months after surgery (*P* < .001) (Table 7). In each studied group, the posterior corneal elevation map was analyzed. After cataract surgery in all patients, we noticed significant changes of the posterior corneal surface elevation map at the periphery in the meridians of 90° to 120°regardless of the surgical technique used. In this location the maximum posterior corneal surface elevation value at the periphery was analyzed. In this paper, we present the results only in relation to the place where we obtained significant values in the results. In each studied group, there was statistically significant difference at each timepoint in term of the posterior corneal elevation map at the periphery assessed within the 90° to 120° meridian range. On the first and on the seventh postoperative day the values in the group A and in the group C were statistically significantly higher than in the group B (*P* < .001). But after 1 month postoperatively the values in the group C were statistically significantly higher than in the group A and in the group B (*P* = .004). The values in the group B and in the group C were statistically significantly higher than in the group A 6 months after surgery (*P* < .001). Postoperative changes in the value of the posterior corneal elevation map at the periphery assessed within the 90° to 120° meridian range comes back the most quickly to the baseline on the first and on the seventh postoperative day in the group B compared to the group A and to the group C (Table 8).

**Table 3:**
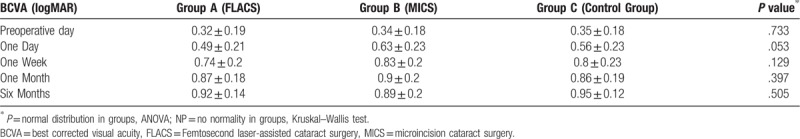
Mean values of best corrected visual acuity in preoperative and postoperative parameters for group A, group B, and group C.

**Table 4:**
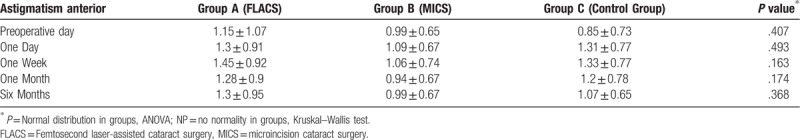
Mean values of astigmatism anterior in preoperative and postoperative parameters for group A, group B, and group C.

**Table 5:**
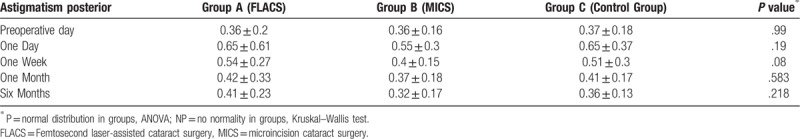
Mean values of astigmatism posterior in preoperative and postoperative parameters for group A, group B, and group C.

**Table 6:**
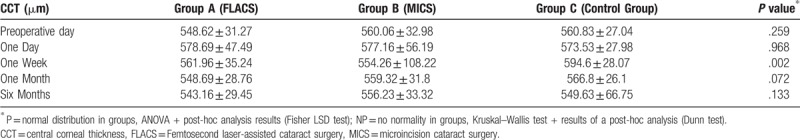
Mean values of central corneal thickness in preoperative and postoperative parameters for group A, group B, and group C.

**Table 7:**
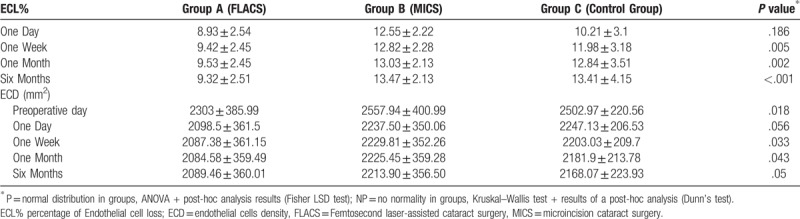
Mean values of percentage of endothelial cell loss and endothelial cells density in preoperative and postoperative parameters for group A, group B, and group C.

**Table 8:**
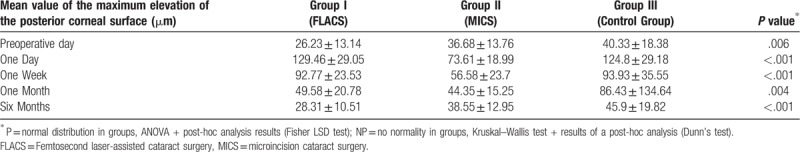
Mean values of the maximum elevation of the posterior corneal surface at the periphery assessed within the 90° to 120° meridian range in preoperative and postoperative parameters for the group A, group B, and group C.

There were no major postoperative complications. There were not any intraoperative miosis. We noted the following postoperative complications that did not affect visual quality: conjunctival redness or hemorrhage, corneal edema, local Descemet membrane detachment. Conjunctival redness or hemorrhage occurred in 3 patients in the group A and there were no observed 1 month after surgery. Conjunctival redness or hemorrhage were no observed in the group B and in the control group. Corneal edema occurred on the 1st day after the procedure in 7 patients in the group A and in 2 patients in the group B. In the group C there was not observed corneal edema after surgery. Corneal edema was not observed in subsequent follow-up visits to the subjects. Local Descemet membrane detachment, which occurred in the first post-operative day in 1 patient in the group Band in the group C, in 3 patients in the group A. Local Descemet membrane detachment was not observed 1 month after surgery and 6 months after surgery. These complications not visible in the slit lamp examination were observed in anterior segment optical coherence tomography (AS-OCT, CASIA II). Complications which occurred are transient and do not affect the final outcome of the treatment.

## 4 Discussion and conclusions

Femtosecond laser-assisted cataract surgery is the newest achievement in cataract surgery, starting from their first clinical usage in 2008. Nowadays patients expect to achieve optimum refractive outcome, more rapid visual rehabilitation, and experience fewer traumas therefore demand for new technologies is still increasing. Such expectations seem to be fulfilled by the femtosecond laser that ensures safety, effectiveness, and repeatability of the treatments performed which has been well documented in the literature.[^26^,^27^,^28^,^29^] There have been many publications on the advantages of femtosecond laser-assisted cataract surgery compared to the conventional phacoemulsification cataract surgery.[^5^,^11^,^12^,^14^,^16^,^20^,^23^,^28^,^30^,^31^]

In our study, we found no statistically significant difference in BCVA on the first day, on the seventh day, 1 month and 6 months after surgery between the three studied groups. Our results coincide with many publications. Popovic et al made a meta-analysis 15 randomized controlled trials and 22 observational cohort studies that included 14,567 that demonstrated no statistically significant difference was detected between FLACS and manual phacoemulsification in uncorrected and corrected visual acuity.^[32]^ In the Cochrane review by Day et al that included 16 randomized controlled trials (RCTs) there no found any important difference in postoperative visual acuity between FLACS and CPS. The mean difference (MD) was −0.03 logMAR in corrected distance visual acuity (CDVA) 6 months after surgery in favor of the laser cataract surgery but it was considered clinically insignificant.^[33]^ Other authors in their research also no reported differences in postoperative BCVA.[^34^,^35^] Shentu et al in their report pooled results of the literature and indicated no significant differences between the C-MICS group and the C-SICS group on postoperative BCVA similarly to our study.^[36]^ Wilczyński et al also reported no statistically significant differences in BCVA between B-MICS vs standard CPS.^[37]^

The application of ultrasound energy is an essential step in phacoemulsification surgery but not without its risk. In literature, the relationship between high power and a long time of used ultrasound and their destructive effect on corneal endothelium was proven.^[9]^ In our study analyzed groups differed statistically significantly in ECL%. The percentage of loss of corneal endothelial cell density after the operative procedure in the group A is statistically significantly lower compared to cataract removal in group B and in the group C executed by standard CPS. We would like to notice that in the presented study there is a slightly lower but statistically insignificant LOCS III grade in the group A compared to the group B and to the group C that can affect the results of ECL after cataract surgery. We would like to emphasize, although this is statistically insignificant difference, it can make change of ECL after surgery in compared studied groups. We realize that a small study group is a limitation in our study which may have an effect on some of the final results. In our opinion there are necessary further investigations in the larger studied groups with a longer follow-up period. Our data are in accordance with previous publications demonstrating that FLACS is less traumatic to the corneal endothelium than CPS.[^11^,^30^,^38^] Similar to our study Abell et al in their study revealed a significant reduction in endothelial cell lost and in early postoperative corneal edema in FLACS group compared to CPS.^[13]^ Conrad-Hengerer et al in their prospective randomized study as well as Krarup et al in and other authors in their studies reported that the endothelial cell loss was lower in the LCS group than in the control CPS group.^[^ [^11^,^12^,^13^,^39^,^40^,^41^,^42^,^43^,^44^] In contrary to our study Bascaran et al did not found significant differences between the two groups in endothelial cell density loss and CCT either.[^45^,^46^] In turn, in the group B and in the group C there were no statistically significant differences in the loss of endothelial cells between studied groups. Similarly to our study Mencucci et al and Wang et al were no found significant differences in corneal endothelial cell loss or endothelial morphology between MICS and standard incision techniques.[^47^,^48^] Wilczyński et al reported the same that ECL in MICS group is similar to standard CPS group.^[37]^ On the other hand Shentu et al and Crema et al concluded that in MICS group induced a higher ECL% in comparison to SICS group.[^36^,^49^]

There was a statistically significant difference in CCT between 3 groups on the seventh postoperative day. The values in the group C were higher than in the group A and in the group B, but in 6 months follow up it was not statistically different at last postoperative control between studied groups. Wang et al reported no statistical significant differences in CCT between MICS and SICS.[^46^,^48^] Other authors in their studies also were no noted any differences in CCT between studied groups.[^34^,^40^,^45^,^50^]

On the other hand, the irrigation solution volume used during the entire procedure should be taken into account. In the group B and in the group C we reported a statistically significant higher usage of volume intraoperative irrigation solution compared to FLACS group consumption which may have an impact on increase of hydrodynamic flow and turbulence in the anterior chamber potentially damaging endothelial cells.^[51]^ This result might be considered in terms of a cost-effectiveness benefit for surgery. Comparable to our results Cavallini et al in their study also found statistically significantly reduced BSS usage in the FLACS group.^[50]^ Four RCTs, representing 288 eyes, published in the report by Shentu et al showed that there was no significant differences between C-MICS and C-SICS groups in the context of BSS use.^[36]^.

In our study, there was no statistically significant difference in the pre- and postoperative changes in the terms of the central anterior and posterior astigmatism between studied groups. Other authors in their studies also no observed significant changes concerning astigmatism between pre- and postoperative values.^[50]^ We observed significant change in the posterior elevation map at the periphery assessed within the 90° to 120°meridian range. On the first and seventh postoperative day the values in the group A and in the group C were statistically significantly higher than in the group B. The difference in the posterior elevation map at the periphery was not significant on seventh day in the group B, but still significant up to one month in the group A and in the group C. Postoperative change in the value of the posterior corneal elevation map comes back the most quickly to baseline on day 1 and day 7 for the group B treatment, compared to the group A and to the group C. These results do not affect the final visual acuity. There is no statistically significant difference in BCVA between patients in the studied groups. However, we suspect that these results may affect the quality of vision in patients after surgery, especially in the early postoperative period but this requires further functional testing. The 2.4 mm incisions have a higher impact on the posterior corneal surface elevation at the periphery in the main corneal incision meridian than 1.8 mm incision. We can presume that the reduction in the size of the corneal incision reduces the elevation of the posterior surface of the cornea, which in clinical practice may translate into faster healing of the cornea and its return to the pre-operative state. This described difference has actually an unknown effect on the healing process after cataract surgery. To confirm this hypothesis, comparative studies on a larger group of patients are required.

Our results show that effective phacoemulsification time (EPT) was statistically significantly lower in the group A compared to the standard CPS in the group B and in the group C. We suppose that is due to prefragmentation of the nucleus into larger pieces with the use of femtosecond laser usage. In our study total surgery time was statistically significantly higher in the group A compared to the group B and to the group C. In our opinion that it is due to the docking procedure with the femtosecond laser at the beginning of the surgery. Popovic et al analyzed extensive literature research in which reported no statistically significant difference between FLACS and manual phacoemulsification in total surgery time, but they reported that effective phacoemulsification time (EPT) was significantly lower in laser cataract surgery in eight out of 8 analyzed studies what is similar to the results in our study.^[32]^ There are many publications where reported reduction of EPT in FLACS group compared to CPS group.[^12^,^13^,^40^,^42^,^43^,^44^] Comparing the group B to the group C in our study we no reported difference in EPT and total surgery time between analyzed groups similarly to report analyzed by Shentu et al analyzed 5 RCTs, representing by 244 eyes and reported no significant difference was found between the C-MICS group and the C-SICS group in EPT. Similarly to our results Wang et al in their meta-analysis included eleven RCT studies reported no statistically significant differences were observed in EPT between C-MICS compared to standard-incision phacoemulsification.^[48]^ They also analyzed 6 RCTs, representing 508 eyes, reported mean surgery time and results showed no statistical differences between the C-MICS group and the C-SICS group.^[36]^

We did not record any intraoperative and postoperative huge complications that could affect the final outcome of the treatment. Postoperative complications which occurred in 6 months follow-up period were following: transient corneal edema, local Descemet membrane detachment at the area of the corneal incision that was seen in the AS-OCT examination performed and was not seen in the slit lamp examination. Thus, the corneal morphology and topography changes after phacoemulsification surgery not visible on the slit lamp examination could be monitored with AS-OCT. Similarly, to the other publications in our study there were not any intraoperative miosis. That is due to the low-energy laser system that results in less inflammation with lower level of prostaglandins in the anterior chamber.[^52^,^53^]

Recently, 2 major meta-analyses appeared in the literature, which give proof that both techniques: FLACS and CPS, are highly effective and safe, and the differences in some parameters are small or non-existent. Moreover, there was found published clinical trials to be at an unclear or high risk of bias.[^32^,^33^] Although femtosecond laser-assisted cataract surgery (FLACS) does not seem to show any significant difference with respect to refractive and visual outcomes as compared to standard phacoemulsification, FLACS introduce advantages in terms of higher predictability, safety and precision.[^22^,^32^,^33^] The economic aspect is also important. The high cost of surgery is a barrier for many centers and patients. There is still no uniform consensus regarding the benefits and disadvantages of FLACS compared to the standard phacoemulsification cataract surgery and it is constantly discussed whether the femtosecond laser will become the standard method for cataract surgery in the future or will not.[^54^,^55^]

To conclude the results of this prospective comparative study revealed that both surgery technique FLACS as well as MICS are safe and effective compared to the standard phacoemulsification by 2.4 mm corneal incision. There no revealed statistically significant differences in terms of visual acuity, astigmatism, central corneal thickness between studied groups in 6 months follow-up. However, in this small prospective comparative study FLACS was associated with significantly reduced in effective phacoemulsification time, BSS usage, decreased the amount of corneal endothelial cell loss compared with both MICS (group B) and conventional phacoemulsification- SICS (group C) although the total surgery time was higher in the FLACS group (group A). Furthermore, postoperative change in the value of the posterior corneal elevation map at the periphery assessed within the 90° to 120°meridian range comes back the most quickly to baseline on day 1 and day 7 for the group B (MICS group) compared to the group A (FLACS group) and to the group C (control group), what was statistically significant. The results of our research are consistent with existing publications. We cannot rule out, however, the small size of the study group in our study, that may affect the presented results and statistical significance. We realize there are some limitations in this study such it is a non-blinded study with a small group size. Up until now available publications fail to show coherent evidence that one of the studied surgical techniques are better than the others. We hope our paper will help to define the real effect of FLACS on the selected parameters of the anterior segment of the eye. Although in our opinion there are still necessary further investigations and analysis of each parameter of the eye's tissues and especially its clinical effects in the larger studied groups and longer follow-up periods.

## Author contributions

**Conceptualization:** Edyta Chlasta-Twardzik, Edward Wylęgała.

**Data curation:** Edyta Chlasta-Twardzik, Anna Nowińska.

**Formal analysis:** Edyta Chlasta-Twardzik.

**Funding acquisition:** Anna Nowińska.

**Investigation:** Edyta Chlasta-Twardzik.

**Methodology:** Edyta Chlasta-Twardzik.

**Project administration:** Edyta Chlasta-Twardzik.

**Resources:** Edyta Chlasta-Twardzik.

**Software:** Edyta Chlasta-Twardzik.

**Supervision:** Edward Wylęgała.

**Validation:** Edyta Chlasta-Twardzik.

**Visualization:** Edyta Chlasta-Twardzik.

**Writing – original draft:** Edyta Chlasta-Twardzik.

**Writing – review & editing:** Anna Nowińska, Edward Wylęgała.
